# Genome-Wide Signatures of Transcription Factor Activity: Connecting Transcription Factors, Disease, and Small Molecules

**DOI:** 10.1371/journal.pcbi.1003198

**Published:** 2013-09-05

**Authors:** Jing Chen, Zhen Hu, Mukta Phatak, John Reichard, Johannes M. Freudenberg, Siva Sivaganesan, Mario Medvedovic

**Affiliations:** 1 Laboratory for Statistical Genomics and Systems Biology, Department of Environmental Health, University of Cincinnati College of Medicine, Cincinnati, Ohio, United States of America; 2 Department of Environmental Health, University of Cincinnati, Cincinnati, Ohio, United States of America; 3 Toxicology Excellence for Risk Assessment (TERA), Cincinnati, Ohio, United States of America; 4 Mathematical Sciences Department, University of Cincinnati, Cincinnati, Ohio, United States of America; University of Toronto, Canada

## Abstract

Identifying transcription factors (TF) involved in producing a genome-wide transcriptional profile is an essential step in building mechanistic model that can explain observed gene expression data. We developed a statistical framework for constructing genome-wide signatures of TF activity, and for using such signatures in the analysis of gene expression data produced by complex transcriptional regulatory programs. Our framework integrates ChIP-seq data and appropriately matched gene expression profiles to identify True REGulatory (TREG) TF-gene interactions. It provides genome-wide quantification of the likelihood of regulatory TF-gene interaction that can be used to either identify regulated genes, or as genome-wide signature of TF activity. To effectively use ChIP-seq data, we introduce a novel statistical model that integrates information from all binding “peaks” within 2 Mb window around a gene's transcription start site (TSS), and provides gene-level binding scores and probabilities of regulatory interaction. In the second step we integrate these binding scores and regulatory probabilities with gene expression data to assess the likelihood of True REGulatory (TREG) TF-gene interactions. We demonstrate the advantages of TREG framework in identifying genes regulated by two TFs with widely different distribution of functional binding events (ERα and E2f1). We also show that TREG signatures of TF activity vastly improve our ability to detect involvement of ERα in producing complex diseases-related transcriptional profiles. Through a large study of disease-related transcriptional signatures and transcriptional signatures of drug activity, we demonstrate that increase in statistical power associated with the use of TREG signatures makes the crucial difference in identifying key targets for treatment, and drugs to use for treatment. All methods are implemented in an open-source R package *treg*. The package also contains all data used in the analysis including 494 TREG binding profiles based on ENCODE ChIP-seq data. The *treg* package can be downloaded at http://GenomicsPortals.org.

## Author Summary

Knowing transcription factors (TF) that regulate expression of differentially expressed genes is essential for understanding signaling cascades and regulatory mechanisms that lead to changes in gene expression. We developed methods for constructing gene-level scores (TREG binding scores) measuring likelihood that the gene is regulated based on the generative statistical model of ChIP-seq data for all genes (TREG binding profile). We also developed methods for integrating TREG binding scores with appropriately matched gene expression data to create TREG signatures of the TF activity. We then use TREG binding profiles and TREG signatures to identify TFs involved in the disease-related gene expression profiles. Two main findings of our study are: 1) TREG binding scores derived from ChIP-seq data are more informative than simple alternatives that can be used to summarize ChIP-seq data; and 2) TREG signatures that integrate the binding and gene expression data are more sensitive in detecting evidence of TF regulatory activity than commonly used alternatives. We show that this advantage of TREG signatures can make the difference between being able and not being able to infer TF regulatory activity in complex transcriptional profiles. This increased sensitivity was critically important in establishing connections between disease and drug signatures.

## Introduction

The specificity of transcriptional initiation in the genomes of eukaryotes is maintained through regulatory programs entailing complex interactions among transcription factors (TF), epigenetic modifications of regulatory DNA regions and associated histones, chromatin-remodeling proteins, and the basal transcriptional machinery [1]. High-throughput sequencing of immuno-precipitated DNA fragments (ChIP-seq) provides means to assess genome-wide expression regulatory events, such as TF-DNA interactions [2]. Sophisticated statistical methodologies have been developed for identifying TF binding events in terms of “peaks” in the distributions of ChIP-seq data [3]–[8]. The evidence provided by ChIP-seq binding data that a gene's expression is regulated by a TF is a function of the number of peaks, their intensity and proximity to the transcription start site (TSS) [9]. Furthermore, binding of a transcription factor in a gene's promoter alone does not always result in transcriptional regulation. In the case of highly studied pleiotropic regulator ERα, transcriptional regulation depends on the presence of specific co-factors as well as on the type of activating ligand [10], [11]. Therefore, the identification of true regulatory TF-gene relationships requires per-gene summaries/scores measuring the totality of the evidence in ChIP-seq data, integrated with measurements of gene expression levels.

Current approaches to summarizing binding peaks in order to correlate TF binding with transcriptional changes range from simple summaries in proximal gene promoter (e.g. maximum peak height within a narrow region around the promoter) [12]–[14] to weighted sums of peak heights where weights are inversely proportional to the distance of the peak to the gene's TSS [9], [15]. Currently used distance-based weights are dependent on TF-specific tuning constants established through ad-hoc examination of the distribution of the peaks [9], [12], [13].

Dysregulation of transcriptional programs is intimately related to the progression of cancer [16], [17] and other human diseases [18], [19]. Modulating the behavior of specific TFs is a popular strategy for developing new disease treatments [20]–[23]. Genome-wide transcriptional profiles associated with a disease phenotype provide indirect evidence of TF involvement in the etiology of the disease. The most common strategy of implicating TF involvement is by computational analysis of genomic regulatory regions of differentially expressed genes [24]–[27]. However, such strategies are not effective when the search needs to include distant enhancers and when concurrent activity of multiple regulatory programs lead to “messy” transcriptional signatures. ERα-driven proliferation is one such case where the involvement of ERα regulatory program has been difficult to identify in resulting transcriptional profiles using the DNA binding motif analysis [27].

We have developed a comprehensive statistical framework for assessing True REGulatory (**TREG**) TF-gene interactions by integrated analysis of ChIP-seq and gene expression data. In the first step we introduce a novel two-stage mixture generative statistical model for summarizing “peaks” within 2MB window centered around a gene's TSS. Fitting this two-stage model yields scores and associated probabilities of regulation based on ChIP-seq data alone (ie TREG binding profile). We show that our approach produces effective summaries for a TF with binding sites clustered in close proximity of TSS (E2F1) and a TF known to exhibit regulation through binding to distant enhancers (ERα).

In the second step we integrate the TREG binding profile with a differential gene expression profile to create an integrated TREG signature of TF regulatory activity. We use TREG signatures to detect faint signals of ERα regulation in “messy” transcriptional signature, and demonstrate how such analysis can yield better drug candidates than simply correlating transcriptional signatures of the disease and the drug activity [28]–[30].

## Results

**Figure 1:**
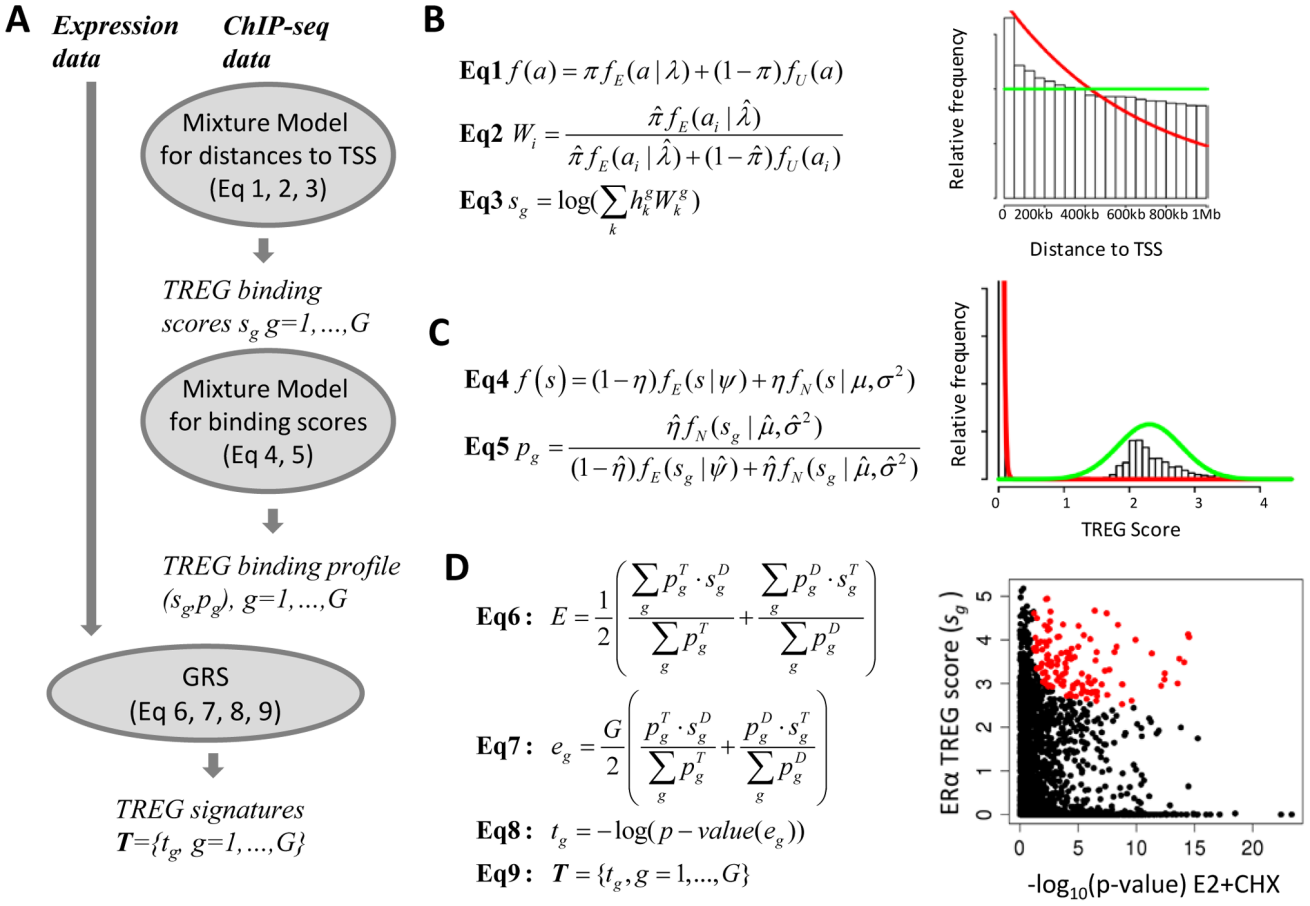
An overview of the TREG framework and statistical models for constructing TREG signatures. **A**) “Peaks” extracted from ChIP-seq binding data and differential gene expression profile that eventually yield the integrated TREG signature of TF activity. **B**) The exponential-uniform mixture module describing the distribution of functional and non-functional “peaks” in ChIP-seq TF-gene binding data. **C**) The exponential-normal mixture module describes the distribution of TREG binding scores for regulated and non-regulated genes. **D**) The Generalized Random Set (GRS) methodology for integrating ChIP-seq and differential gene expression data.

An overview of the TREG framework is shown in Fig. 1. We start with “peaks” extracted from ChIP-seq binding data and differential gene expression profile that eventually yield the integrated TREG signature of TF activity (Fig. 1A). The foundation of the TREG framework consists of two statistical mixture modules. **The first mixture model** describes the distribution of functional and non-functional “peaks” in ChIP-seq TF-gene binding data (Fig. 1B). Based on this model, we derive the TF-specific distance weights and construct gene-level binding scores (TREG binding scores) measuring the likelihood that a gene is regulated by the given TF. **The second mixture model** describes the distribution of TREG binding scores for regulated and non-regulated genes (Fig. 1C). This second model provides us with gene-level probabilities that genes are regulated by a specific TF based on the ChIP-seq data alone. TREG binding scores and associated gene-level probabilities for all genes make up the **TREG binding profile**. The TREG binding profile and differential gene expression profiles are integrated using Generalized Random Set (GRS) methodology [31] to produce an integrated genome-wide **TREG signature** of the TF activity (Fig. 1D). The TREG signature of ERα is used to demonstrate involvement of its regulatory activity in complex transcriptional profiles and to mine Connectivity Map Data for inhibitors of its activity.

### The first mixture module: Deriving gene-specific TREG scores (Fig. 1B)

We assume that observed peaks consist of two populations: **Functional peaks** that are more likely to occur closer to TSS and whose distance to TSS is distributed as an exponential random variable; and, **Non-functional peaks** that are randomly occurring throughout the 2 million base pair genomic region centered around the TSS, and whose distances to TSS are distributed as a uniform random variable. The distances to TSS of all peaks are then distributed as a mixture of the exponential and the uniform distribution (Fig. 1, **Eq1**), where π is the proportion of functional peaks among all observed peaks. We define the **TREG binding score** for gene g as the logarithm of the weighted average of peak intensities, using the probability of the peak belonging to the population of “functional peak” as weights (Fig. 1 **Eq3**).

### TREG binding scores provide an effective gene-level measure of TF regulation

We assessed the effectiveness of the TREG binding score by comparison to the simple scoring method based on the maximum peak intensity (MPI) within a window of specific size around TSS. The two types of scores were evaluated by comparing the enrichment of genes with high evidence of TF binding among genes differentially expressed in appropriately matched experiments. For gene expression data, we identified genes differentially expressed (two-tailed FDR<0.01) 24 h after treating MCF-7 cell line with estradiol (**E2**) with and without pre-treating the cell line with Cycloheximide (CHX) [27]. CHX is an inhibitor of protein biosynthesis in eukaryotic organisms. Treatment with E2 after pre-treatment with CHX (**E2+CHX**) resulted in differential expression of genes presumed to be directly regulated by ERα; whereas after E2 treatment without CHX, the majority of differentially expressed genes were secondary target genes functionally enriched for cell-cycle genes and reflective of the rapid proliferation resulting from the E2 treatment [27]. For the TF binding data, we used ChIP-seq analysis of the key proliferation regulator E2f1 in growing mouse embryonic stem (ES) cells [32], and ERα binding 1 h after treating MCF-7 cells with estradiol [10]. ChIP-seq data at 1 h hour after treatment with E2 is correlated with gene expression changes 24 h after treatment because of the expected time-delay between ERα binding to a gene promoter and the observable change in the gene's expression level.

**Figure 2:**
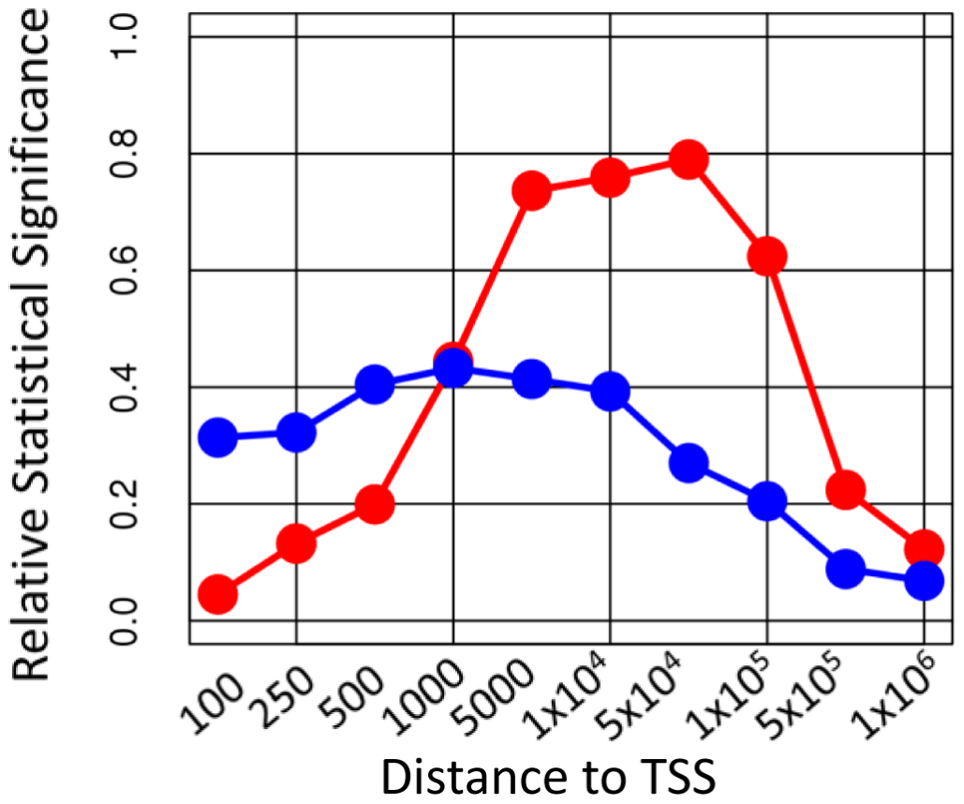
Relative statistical significance of the association between ChIP-seq and differential gene expression data for different window sizes. The ratio of −log_10_(p-value of enrichment) of differentially expressed genes (FDR<0.1) among genes with high MPI scores, and −log_10_(p-value of enrichment) of differentially expressed genes among genes with high TREG binding scores. The ratios related to E2f1 ChIP-seq data and E2 differential gene expression profile are represented by the blue line. The ratios related to ERα ChIP-seq data and are represented by the red line. Ratios smaller than 1 indicate higher significance of enrichment when using TREG scores, as opposed to maximum peak height within the given window.

Among differentially expressed genes, enrichment of genes with high TREG binding scores was statistically significant for both E2F1 and ERα in both experiments (Table 1). Fig. 2 shows the relative levels of enrichment for maximum peak intensity (MPI) score over the range of window sizes around TSSs in comparison to the TREG binding score. Simple MPI scores never attain the level of statistical significance of enrichment attained by TREG binding scores. Furthermore, the performance of the simple score is heavily dependent on the specific size of the window used, and expectedly, the optimal windows are TF–specific. The optimal window size for E2f1 and ERα is around 1 kb and 50 kb respectively, with maximum statistical significance of enrichment attained for the simple score reaching 42% and 80% of the TREG binding score significance, respectively. Similar results were obtained using unweighted sum and linear-weighted sum of TF binding peak intensity scores (supplementary results in Text S1 and Fig. S1). This indicates that TREG binding scores not only provide the best correlation with expression changes, but they also obviate the need of knowing the right window size to use in deriving the summary measure of TF binding. The calculation of TREG binding scores does not include any free parameters that need to be specified in ad-hoc fashion, such as the length of the genomic region around TSS for simple scores, or the ad-hoc weighting parameters used in similar scores before [9], [15].

**Table 1:** Statistical significance of LRpath enrichment of genes with high TREG binding scores for E2f1 and ERα among differentially expressed genes (two-tailed FDR<0.01) for E2 and E2+CHX differential gene expression profiles.

Transcription Factor	E2	E2+CHX
**E2f1**	7.0×10^−79^	1.6×10^−7^
**ERα**	6.2×10^−13^	1.0×10^−76^

The analysis was performed using LRpath methodology.

### The second mixture module: Gene-level ChIP-Seq binding probability mixture model

Having constructed gene-specific TREG binding score, our goal was to estimate gene-level probabilities of “functional interaction” between a TF and a gene based on these scores. The histogram of the TREG binding scores (Fig. 1C) clearly shows two populations of TREG binding scores. One population with a majority of TREG binding scores being close to zero, representing genes with low likelihood of functional TF-gene interaction, and the other populations with TREG binding scores distributed in bell-shaped form around the mean slightly higher than 2, representing functional interactions. Therefore, we assume that TREG binding scores come from two populations: Scores significantly greater than zero representing functional TF-gene interactions which are distributed as a Normal random variable; and, scores close to zero representing non-functional interactions which are distributed as an exponential random variable. Assuming that the proportion of TREG binding scores corresponding to functional interactions is η, the distribution of all TREG binding scores is a mixture of Normal and exponential probability distribution functions (Fig. 1 **Eq4**). The probability that a TREG binding score for gene *g* (*S_g_*) is functional is defined as the probability of *S_g_* belonging to the normal component (Fig. 1 **Eq5**). The set of TREG binding scores and associated probabilities of the score indicated functional TF-gene interaction for all genes in the genome (S_g_, p_g_), g = 1,…,G, is the **TREG binding profile**.

### Integrating TREG binding profile and differential gene expression to identify regulated genes

Identifying genes that both have high probability of “functional” TF binding and are differentially expressed is complicated by the need to set arbitrary thresholds for statistical significance. We have previously developed a method, based on the Generalized Random Set (GRS) analysis that obviates the need for such thresholds when assessing concordance of two differential gene expression profiles [31]. Here we apply the GRS framework to assess the concordance between the TREG binding profile and the differential gene expression profile (Fig. 1 **Eq6**) (details in Text S1), and to identify genes with statistically significant concordance. The results (Table 2) of the analysis generally followed the results based on designating differentially expressed genes (Table 1) with the levels of statistical significance being orders of magnitude higher in the GRS concordance analysis. We demonstrate that GRS is producing expected distribution of p-values under the null hypothesis by systematically examining empirical cumulative distribution functions (ECDFs) of p-values after randomly permuting gene labels in TREG binding profile before GRS analysis (supplementary results in Text S1, Fig. S2). We also compared the results of GRS analysis with the thresholding approach based on TREG binding probability where gene was placed in the “regulated” group if the corresponding TREG probability (p_g_) was greater than 0.95. Results were similar to the GRS analysis (supplemental results Text S1). However, we also show that in the situations when binding signal is relatively “faint”, GRS is likely to outperform thresholding approach (Text S1, Fig. S3). Since these are situations in which the method of concordance analysis will make the difference, the GRS is still likely the better default choice for performing the concordance analysis.

**Table 2:** P-values for TREG concordance analysis between TREG binding profiles (E2f1 and ERα) and differential gene expression profiles (E2 and E2+CHX).

Transcription Factor	E2	E2+CHX
**E2f1**	1.1×10^−162^	2.2×10^−13^
**ERα**	1.2×10^−28^	9.1×10^−124^

Finally, we integrate at the gene level TREG binding profiles with differential gene expression profiles as the contribution of an individual gene to the overall concordance in the GRS concordance statistics *e_g_* (Fig. 1 **Eq7**). The statistical significance of gene-level GRS statistics is assessed by associated resampling-based p-values (see methods) which define gene-specific **TREG concordance scores** (*t_g_*, Fig. 1, **Eq8**). The vector of such scores for all genes represents the **TREG signature of TF activity** (Fig1 **Eq9**).

### The power of TREG binding profiles and TREG signatures in identifying TF targets

**Figure 3:**
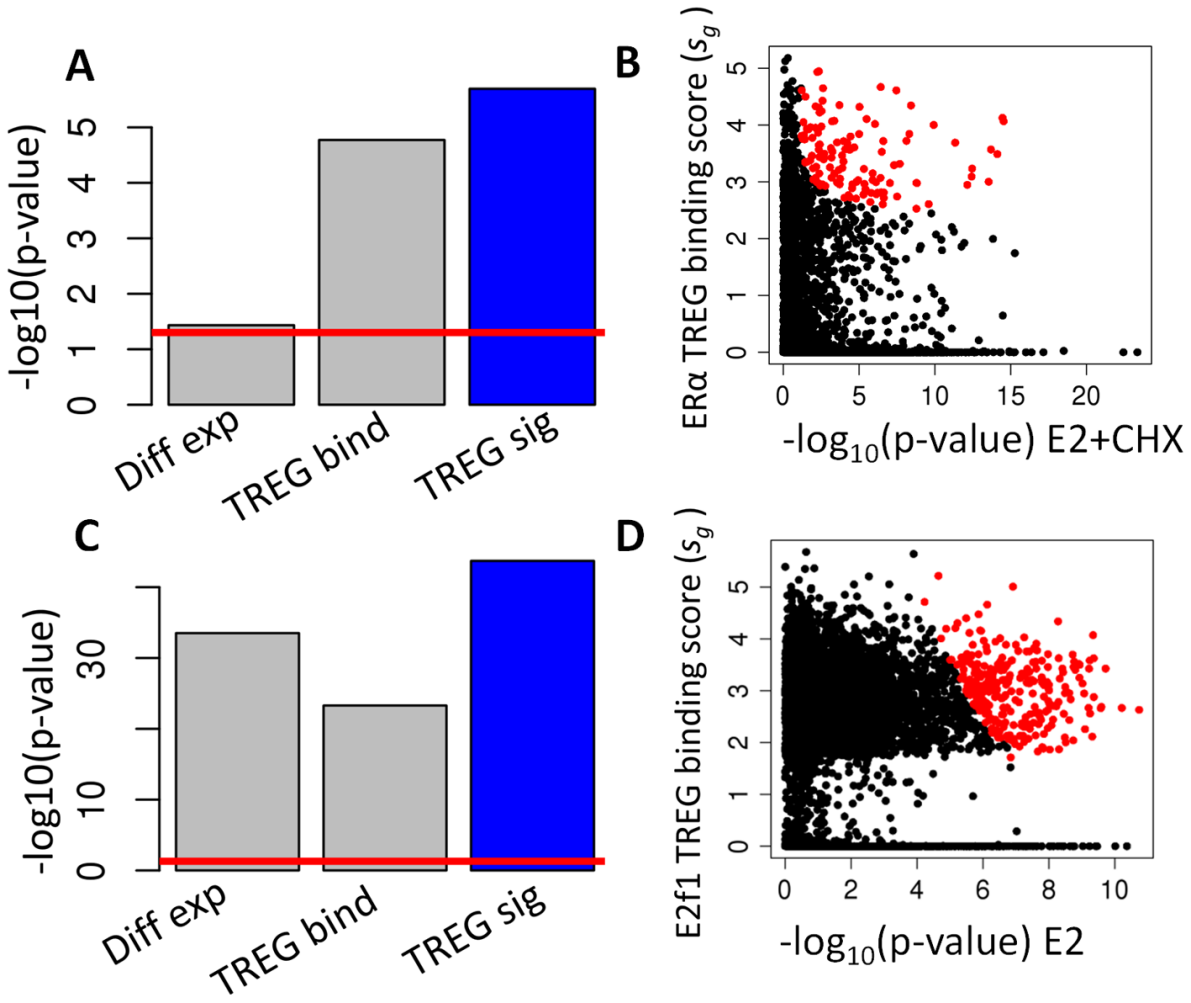
Pinpointing regulated genes by integrating binding and differential gene expression data. **A**) Statistical significance of enrichment of computationally predicted ERα targets from MSigDB database using the E2+CHX differential gene expression profile, (Diff Exp), ERα TREG binding scores (TREG bind) and the TREG signature integrating expression and ChIP-seq data (TREG sig) (the red line indicate p-value of 0.05). **B**) The scatter plot of TREG binding scores against the statistical significance of differential gene expression. The red points indicate genes with statistically significant TREG concordance scores (t_g_>−log_10_(0.01)). The red points were overlaid over the black points which means that all significant points are visible **C**) Statistical significance of enrichment of computationally predicted E2F1 targets from MSigDB database using the E2 differential gene expression profile, (Diff Exp), E2f1 TREG binding scores (TREG bind) and the TREG signature integrating expression and ChIP-seq data (TREG sig) (the red line indicate p-value of 0.05). **D**) The scatter plot of TREG binding scores against the statistical significance of differential gene expression as in **B**.

We examined the ability of TREG binding profiles and TREG signatures to identify genes regulated by ERα and E2f1. Fig. 3A contrasts the statistical significance of the enrichment by the computationally predicted ERα targets from MSigDB database [33] based on E2+CHX differential gene expression profile (Diff Exp), ERα TREG binding scores (TREG bind) and integrated TREG signature (TREG sig). In this setting, MSigDB targets provide a “noisy” gold standard since the perfect gold standard does not exist. While all three data types provided statistically significant enrichment, the integrated TREG signature showed the highest statistical significance of the enrichment. The overall relationship between the TREG binding scores, statistical significance of differential gene expression (−log_10_(p-value) E2+CHX) and the statistical significance of TREG concordance scores (ERα TREG score (*s_g_*)) is shown in Fig. 3B. The “statistically significant” (p-value<0.001) TREG concordance scores (red dots in Fig. 3B) required both, a high TREG binding score and a high statistical significance of differential expression. Similar analysis of the E2f1 TREG signature showed a similar pattern (Fig. 3C and D), although the overall statistical significance of enrichment was much higher for all three data types. These results show that integrated TREG signatures are more informative of the regulatory TF-gene relationships than expression or TF binding data alone. TREG binding scores, gene specific concordance statistic, and TREG concordance scores for all genes are given in the Table S1.

### Functional analysis of ERα and E2F1 TREG signatures

**Figure 4:**
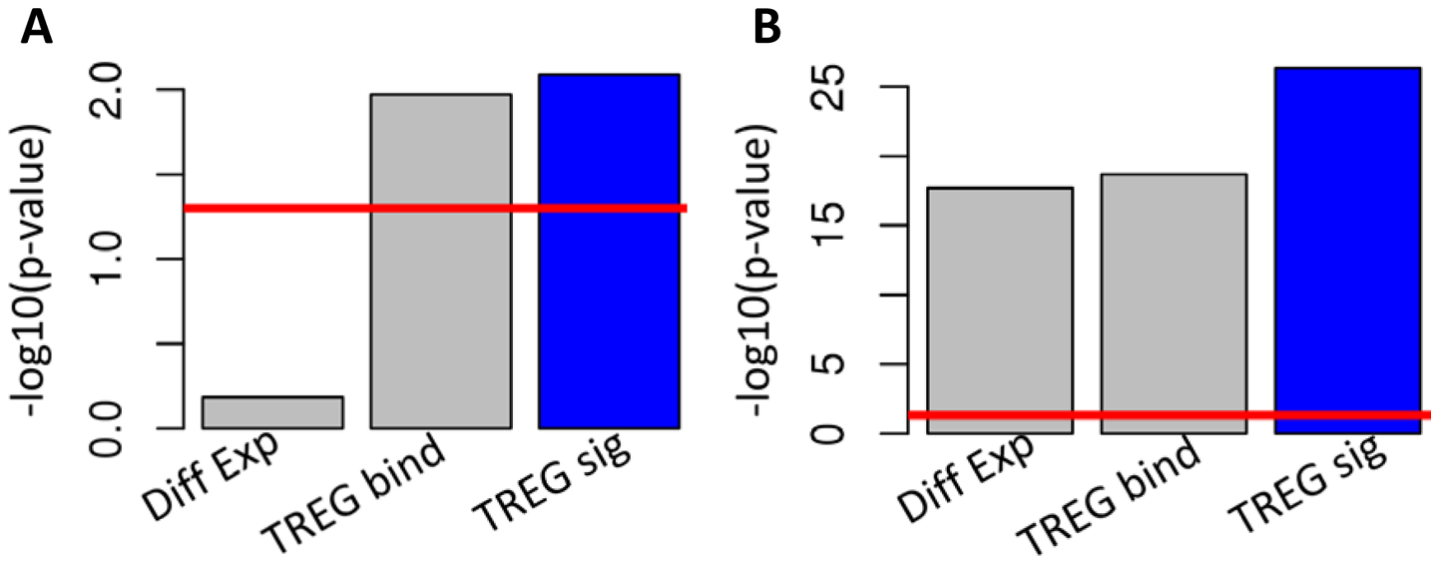
Function of regulated genes. Enrichment of ER*α* and E2F1 targets among genes associated with two prototypical functional categories associated with ER*α* (response to estrogen stimulus) and E2F1 (regulation of mitotic cell cycle) function. **A**) Statistical significance of enrichment of computationally predicted genes associated with “response to estrogen stimulus” using the E2+CHX differential gene expression profile, (Diff Exp), ERα TREG binding scores (TREG bind) and the TREG signature integrating expression and ChIP-seq data (TREG sig) (the red line indicate p-value of 0.05). **B**) Statistical significance of enrichment of computationally predicted genes associated with “regulation of mitotic cell cycle” using the E2 differential gene expression profile, (Diff Exp), E2f1 TREG binding scores (TREG bind) and the TREG signature integrating expression and ChIP-seq data (TREG sig) (the red line indicate p-value of 0.05).

**Figure 5:**
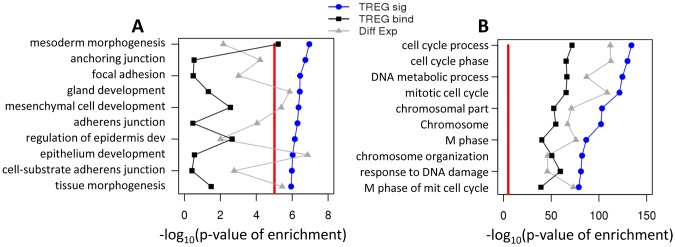
Distinctive functional roles of ERα and E2F1 targets. Top 10 enriched gene lists associated with Gene Ontology terms using the TREG signatures for enrichment analysis. **A**) Gene lists enriched with ERα regulated genes only. **B**) Gene lists enriched with E2F1 regulated genes only.

We further examined ERα and E2F1 TREG signatures to determine molecular pathways and biological processes regulated by these two TFs and to evaluate benefits of such integrated signatures. We assessed the enrichment of genes with high TREG concordance scores in lists of genes related to the prototypical function of ERα and E2F1. For the ERα signature the list consisted of genes associated with the Gene Ontology term “cellular response to estrogen stimulus”, and for the E2F1 with the term “regulation of mitotic cell cycle”. In both cases, integrated TREG signatures showed significantly higher statistical significance of enrichment than either TREG binding scores or differential gene expressions (Fig. 4). Unsupervised enrichment analysis of the two signatures revealed that biological processes specifically associated with ERα signature were related to the development of the mammary gland (Fig. 5A). Moreover, significant associations between ERα-regulated genes and some key developmental processes could not have been established using either TF binding or gene expression alone. Likewise, processes related to mitotic cell cycle were most highly associated with E2f1 signature (Fig. 5B). Results of enrichment analysis for all GO terms are provided in Table S2.

### TREG methodology applied to ENCODE TF binding data

**Figure 6:**
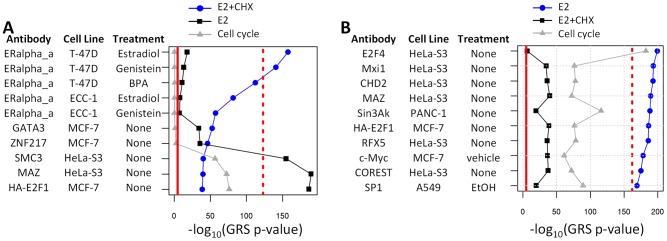
TREG analysis of ENCODE TF binding data. The GRS concordance analysis between E2+CHX and E2 expression profiles and 494 ENCODE TREG profiles. The solid red line indicates statistical significance cut-off and dashed red line indicates the statistical significance attained with ERα and E2f1 TREG profiles we use throughout the paper. Grey symbols/lines in both figures indicate the statistical significance of enrichment of genes with high TREG binding scores among Cell cycle genes. **A**) Top 10 ENCODE TREG binding profiles most concordant with E2+CHX expression profile. All five ERα ENCODE profiles are at the top of the list of the most concordant profiles. **B**) Top 10 ENCODE TREG binding profiles most concordant with E2 expression profile. Profiles of TFs from E2F-family and the c-Myc profile are among the top 10 most concordant signatures.

To assess the reproducibility and specificity of our results, we constructed TREG binding signatures for all 494 TF ChIP-seq datasets in the Genome Browser ENCODE tables [14], [34]. Two gene expression profiles in our analysis (E2+CHX and E2) were then systematically compared with 494 ENCODE TREG binding profiles. Top 10 most concordant profiles are shown in Fig. 6. Results show that ENCODE ERα binding profiles correlates equally well with E2+CHX profile as did our original TREG profile (Fig. 6A). Furthermore, all five ENCODE ERα binding profiles correlated better with E2+CHX profile than any other ENCODE profile. Similarly, ENCODE binding signatures most concordant with E2 profile (Fig. 6B) included E2F4, E2F1 and MYC which are all known to be important cell cycle regulators. The statistical significance of the concordance was again similar to the levels we observed with the E2f1 binding profile in mouse embryonic stem cells. These results indicate that reproducibility of TREG results across different ChIP-seq datasets and its ability to identify key transcriptional regulators for a given profile. Results of the concordance analysis for all ENCODE TREG profiles are in Table S3.

### Finding evidence of ERα activity in complex transcriptional profiles

**Figure 7:**
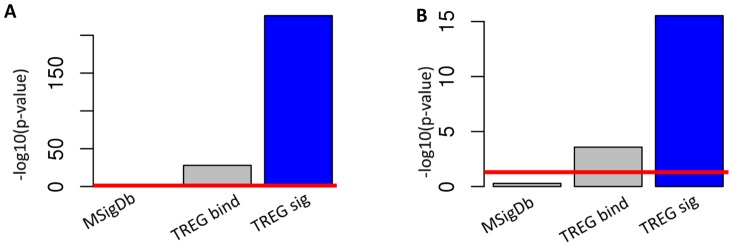
Revealing ERα regulatory activity in complex transcriptional profiles. Contrasting traditional strategy of searching for enrichment of differentially expressed genes among computationally predicted targets (MSigDb) with GRS concordance analysis between differential gene expression profiles with TREG binding profile (TREG bind) and TREG signature (TREG sig). Evidence of ERα regulatory activity is in the form of the statistical significance (−log(p-value)) for the LRpath enrichment analysis (MSigDb) and GRS analysis (TREG bind and TREG sig). The red line indicates the p-value = 0.05. **A**) Evidence of ERα regulatory activity in generating differential gene expression profile of the response of MCF-7 cell line 24 hours after treatment with E2. **B**) Evidence of ERα regulatory activity in generating differential gene expression profile comparing ER− and ER+ breast tumors.

The ultimate goal of the TREG framework is to facilitate identification and characterization of signatures of TFs regulating disease-related differential gene expression profiles (DRGEP). Here we demonstrate the power of TREG signatures and TREG binding scores in elucidating the faint signals of ERα activity in two complex DRGEPs, the response of MCF-7 cell line 24 hours after treatment with E2 [27] and differences between ER− and ER+ breast tumors [35]. In both of these DRGEPs, the signal of direct ERα regulation is “drowned out” by the strong secondary proliferation-related transcriptional signature, and the standard enrichment analysis of computationally predicted ERα targets in MSigDb fails to find evidence of ERα regulation (Fig. 7). However, the GRS concordance analysis with both TREG binding scores and TREG signatures are highly statistically significant, and the TREG signature which integrated binding and transcriptional evidence again shows the highest statistical significance of concordance (Fig. 7). Additional discussion of these results is provided in supplementary results (Text S1).

### ERα activity in perturbation signatures and disease-related gene expression profiles

**Figure 8:**
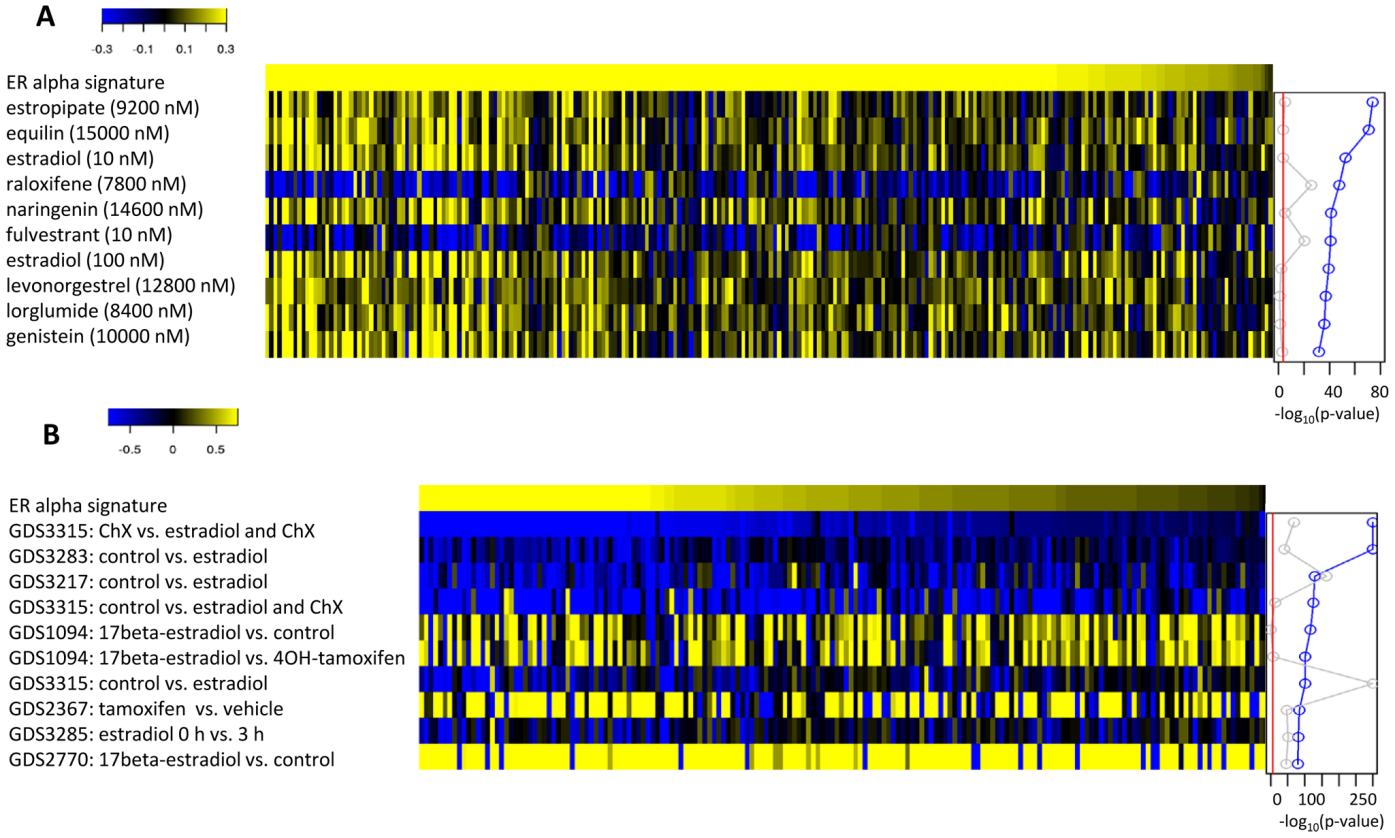
Mining for ERα activity in transcriptional perturbation signatures and disease-related gene expression profiles. Differential gene expression levels (on log_2_ scale) for ERα targets with statistically significant ERα TREG concordance scores (p-value<0.01) in transcriptional signatures with the highest evidence of ERα regulatory activity. The evidence of ERα regulatory activity consisted of the statistical significance of the GRS concordance between ERα TREG signature and the differential gene expression profile (blue line to the right of the heatmap). The ERα signature (top row in each heatmap) represents the differential expression levels in the E2+CHX profile. **A**) Top 10 perturbation signatures with highest evidence of ERα activity among differential gene expression profiles of small drug perturbations in the Connectivity Map dataset. **B**) Top 10 perturbation signatures with highest evidence of ERα activity among differential gene expression profiles of between different sample types in GEO datasets.

We used the ERα TREG signature to mine a collection of differential gene expression profiles in GEO datasets (GDS signatures), and differential gene expression profiles of small drug perturbations (CMAP signatures) [29], for evidence of ERα regulatory activity. Fig. 8 shows differential gene expression levels of top 10 GEO profiles and top 10 drug perturbations based on the statistical significance of the concordance between the ERα TREG signature and each differential gene expression profiles. In both situations the top transcriptional profiles are obviously related to the ERα activity demonstrating the precision of the TREG signature in this setting. Additional results related more specifically to disease-associated GEO profiles are given in the supplementary results (Text S1).

#### Using TREG signatures to connect small molecules, transcription factors, and disease

Using the DRGEP of up-regulated genes in ER+ and ER− breast tumors in comparison to normal mammary epithelium, we mined the Connectivity Map Dataset [29] for putative drugs that could inhibit these signatures. The DRGEPs for ER+ and ER− tumors were created by differential expression analysis between ER+ tumors and the normal breast tissue (ER+ DRGEP) or ER− tumors and normal breast tissue (ER− DRGEP) using the public domain microarray dataset (GSE2740) [36]. We contrasted two distinct strategies. The first approach is the classical CMAP approach of searching for concordance in genes up-regulated in DRGEPs and down-regulated in the drug-signature [28]–[30]. The second approach relied on first elucidating the role of ERα in producing DRGEPs of ER+ and ER− breast cancers, and then searching for drugs that can inhibit ERα signature.

Top five drug candidates for inhibiting ER+ DRGEP, ER− DRGEP and ERα regulatory signature are shown in Table 3. The most highly ranked drugs for both ER+ and ER− breast cancer DRGEPs using the first approach (i.e. direct concordance between DRGEPs and drug transcriptional signatures) included known proliferation inhibitors (e.g. etoposide, pimozide, resveratrol, methotrexate, monobenzone, deferoxamine and trifluridine) [37]–[43].

**Table 3:** Rankings base on the “inhibitory potential” of top 5 CMAP perturbagens for ER+ and ER− DRGEPs, and ERα TREG signature.

Compound	ER+	ER−	ERα
pimozide	1*	10*	128
resveratrol	2*	4*	151
monobenzone	3*	2*	268
chlorpropamide	4*	27*	236
deferoxamine	5*	7*	70
trifluridine	14*	5*	160
methotrexate	19*	3*	181
etoposide	27	1*	59*
tamoxifen	100	110	4*
fulvestrant	112	30	1*
oxaprozin	135	162	5*
raloxifene	266	209	2*
corticosterone	278	303	3*

Stars (*) indicated statistically significant inhibition.

As expected, concordance analysis between ER+ and ER− breast cancer DRGEPs and TREG signatures of ERα and E2F1 activity demonstrated involvement of E2F1 regulation in both DRGEPs (p-value = 7.0×10^−14^ for ER+ and p-value = 1.3×10^−72^ for ER−). This indicates increased proliferation in both types of breast cancers in comparisons to normal tissue. Expectedly, the involvement of ERα regulation was evident only in ER+ DRGEP (p-value = 0.0007), but not in ER− DRGEP (p-value = 0.14), indicating that the increased proliferation is driven by ERα activity only in the ER+ breast cancers. Tamoxifen, raloxifen and fulvestrant were among the top five candidate drugs implicated by their ability to inhibit the ERα activity through the concordance analysis between ERα signature and CMAP data were (Table 3). Tamoxifen and raloxifen are modulators, and fulvestrant is an antagonist of ERα. All three are used in treating ER+ cancers [44]. However, the direct concordance analysis between their transcriptional signatures and ER+ DRGEP would not implicate them as potential treatments. This is most likely due to the subtle ERα signature being overwhelmed by other stronger signals such as the proliferation signature of secondary ERα targets [27].

It is critical to note that alternative approaches to elucidate the role of ERα in producing ER+ breast cancer DRGEP would not have been successful. The standard enrichment analysis against computationally predicted ERα targets fails again to provide any evidence of ERα involvement (p-value = 0.7). Furthermore, even the concordance analysis with TREG binding profile fails to provide statistically significant association in this case (p-value = 0.1). These results demonstrate the sensitivity of the TREG signatures in pinpointing important regulatory mechanisms that can then be exploited in identifying the best drug candidates. In the case at hand, the strategy provided an obvious advantage over the direct strategy of correlating DRGEPs drug transcriptional signatures [28]–[30] to search for drugs that inhibit the global DRGEPs. The improvement in precision resulting from the use of integrated TREG signatures over alternative enrichment strategies that use computationally predicted targets or ChIP-seq data alone, can make a critical difference between the failure or success of such analysis.

## Discussion

The problem of identifying functional TF targets that regulate gene expression, in a specific biological context, requires joint considerations of both TF DNA-binding data and the target gene's expression changes. We described a statistical framework for quantifying the evidence of TF-gene interaction from ChIP-seq data, and integrating them appropriate gene expression data to construct genome-wide signatures of TF activity.

Two main findings of our study are that 1) TREG binding scores derived from ChIP-seq data alone are more informative than simple alternatives that can be used to summarize ChIP-seq data; and 2) TREG signatures that integrate the binding and gene expression data are more sensitive in detecting evidence of TF regulatory activity than available alternatives. We show that this advantage of TREG signatures can make the difference between being able and not being able to infer TF regulatory activity in complex transcriptional profiles. This increased sensitivity also showed to be critical in establishing connections between disease and drug signatures that would not be possible using currently available strategies.

Identifying the role of specific TFs in producing disease-related transcriptional profiles is of vital importance for understanding the molecular mechanisms underlying disease phenotype. Although it is possible to obtain direct measurements of TF activity in disease samples [45], such ChIP-seq profiling is technically challenging and systematic profiling of many different TFs is not feasible. Therefore, the ability to infer the role of a TF from the transcriptional profiles remains challenging. The most common strategy of implicating TF involvement is by computational analysis of genomics regulatory regions of differentially expressed genes [24]–[27], or by searching for enrichment of known targets among differentially expressed genes [46]. Here we present an alternative strategy relying on direct concordance analysis between TREG signatures of TF activity and disease-related transcriptional profiles. When searching for evidence of regulation by the TF with functional binding sites in distant enhancers, such as ERα, and “messy” transcriptional signatures resulting from activity of multiple regulatory programs, our approach dramatically improves the precision of the analysis.

Our results indicate that TREG signatures derived from in-vitro experiments (ERα; MCF-7 cells), and even from a different organism (E2f1; mouse) provide effective means for analyzing transcriptional profiles derived from human tissue samples. This would indicate that TF binding profiles coming from any biological system under which TF shows signs of activity might be sufficiently informative to construct TREG signatures. In this context the recently released ENCODE project data [14], [34] may be turned into a powerful tool for detecting TF activity. As a step in this direction, we have created 494 TREG binding profiles using the ENCODE ChIP-seq data and made it available from the support web-site (http://GenomicsPortals.org). Complementary gene expression data generated by directly perturbing specific TFs, such as shRNA knock-downs and overexpression experiments can be used to construct TREG signatures. For example, transcriptional signatures of such systematic perturbations that is being generated by NIH LINCS project (http://LincsProject.org) could provide complementary transcriptional profiles for ENCODE ChIP-seq data.

Our methods are complementary to methods used to analyze the recently released ENCODE project data [14], [34]. For some experimental conditions, the ENCODE project provides additional data types that can be used in assessing the functionality of TF binding peaks, such as distribution of specific epigenetic histone modifications. For discussion on how to possibly incorporate this additional information within TREG methodology, please see supplemental discussion (Text S1).

Up-regulated expression of proliferation genes is a hallmark of neoplastic transformation and progression in a whole array of different human cancers [47]. While the core transcriptional signature of proliferation is recognizable in a wide range of biological systems and diseases, the events and pathways that drive the transcriptional program of proliferation vary widely. Increased expression of proliferation-associated genes has been associated with poor outcomes in breast cancer patients [48]–[54]. However, the driver mechanisms in many aggressive cancer types are poorly understood. Inhibiting known driver pathways, such as ERα signaling in breast cancer often leads to treatment resistant tumors due to activation of alternative, poorly understood driver pathways [55], [56]. Using the signatures of such “driver events/pathways” we can identify candidate drugs capable of inhibiting them. In our analysis of ERα activity in ER+ breast cancers we showed that such an approach can highlight connections between disease and drug candidates that would be missed by simply correlating disease and drug transcriptional signatures [28]–[30].

## Methods

### Mixture model for summarizing ChIP-seq data and deriving gene-specific TREG scores (Fig. 1B)

We assume that observed peaks consist of two populations: **Functional peaks** that are more likely to occur closer to TSS and whose distance to TSS is distributed as an exponential random variable with the parameter λ; and, **non-functional peaks** that are randomly occurring throughout the 2 million base pair genomic region centered around the TSS, and whose distances to TSS are distributed as a uniform random variable. The distances to TSS of all peaks are then distributed as a mixture of the exponential and the uniform distribution (Fig. 1, **Eq1**), where π is the proportion of functional peaks among all observed peaks, *a* is the distance of a peak to the gene's TSS, 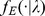 is the probability density function (pdf) of the exponential random variable (rv) with the location parameter *λ*, and 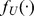 is the pdf of a uniform rv on the interval (−10^6^, 10^6^). We use the standard Expectation-Maximization (EM) algorithm [57] to estimate the parameters of this mixture model (π,λ) for each TF. Given the estimates 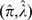 we calculate the posterior probability for peak *i* with distance *a_i_* from a TSS to belong to the population of “functional peaks” (Fig. 1 **Eq2**). Suppose now that for a gene *g*, *n_g_* is the number of peaks within the 1MB window around its TSS (1MB upstream to 1MB downstream), 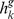 is the peak intensity (ie, the maximum number of overlapping reads over all positions within the peak), and 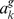 is the distance to TSS of the *k* ^th^ such peak (*k* = 1,…,*n_g_*). We define the **TREG binding score** for the gene *g* as the logarithm of weighted average of peak intensities, using the probability of the peak belonging to the population of “functional peak” (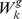) as the weight (Fig. 1 **Eq3**).

### Gene-level ChIP-Seq binding probability mixture model

We assume that TREG binding scores come from two populations: Scores significantly greater than zero representing functional TF-gene interactions which are distributed as a Normal random variable; and, scores close to zero representing non-functional interactions which are distributed as an exponential random variable (histogram in Fig. 1B). Assuming that the proportion of TREG binding scores corresponding to functional interactions is *η*, the distribution of all TREG binding scores is a mixture of Normal and exponential probability distribution functions (Fig. 1 **Eq4**), where *S* is the TREG binding score, 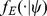 is pdf of the exponential random variable with the location parameter *ψ*, and 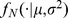 is the pdf of a Normal random variable with mean *μ* and variance *σ^2^*. We again use the standard EM algorithm to estimate the parameters of this mixture model (*η, ψ, μ, σ^2^*) for each TF. Given the estimates 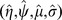, the probability of a TREG binding score for gene *g* (*S_g_*) being functional is defined as the probability of *S_g_* belonging to the normal component (Fig. 1 **Eq5**). The set of TREG binding scores and associated probabilities of the score indicated functional TF-gene interaction for all gene in the genome (S_g_, p_g_), g = 1,…,G, is the **TREG binding profile**. Additional discussion of motivations for the choice of specific distributions is provided in supplemental methods (Text S1).

### EM algorithm

Details of the EM algorithm are provided in supplemental methods (Text S1).

### LRpath enrichment analysis

The enrichment of genes with high TREG and MPI scores among differentially expressed genes (Table 1, Fig. 2) was performed using the logistic regression-based LRpath methodology [58]. LRpath does not require thresholding on binding scores but uses such scores as the continuous variable that explains the membership of a gene in the “differentially expressed” category. Similarly, LRpath was used to analyze enrichment of differentially expressed genes among genes associated with GO terms in Fig. 5 and 6.

### Integrating TREG binding profile and differential gene expression to identify regulated genes

When performing concordance analysis between TREG binding profiles and the two differential gene expression profiles of interest (E2+CHX and E2) (Table 2) and constructing TREG signatures in Fig. 4,5, and 6, we used two-tailed p-values not distinguishing between induction and repression activity. When comparing TREG signatures with other DRGEPs (Table 3, and Fig. 7 and 8), we account for directionality of gene expression changes by using single-tailed p-values for increase in gene expression. This is necessary to account for the directionality of the concordance between the TREG signature and the DRGEPs. The ERα TREG signatures for this analysis was constructed by the GRS concordance analysis (Fig. 1D) between ERα TREG binding profile and the single tailed p-values for statistically significant up-regulation of gene expression after E2+CHX treatment of MCF-7 cell line. The genes used for plotting heatmaps in Fig. 8 were then selected based on the gene-specific p-values of concordance (p-value(*e_g_*), Fig. 1D) being <0.001 (Table S5). The concordance between this ERα TREG signature, and GEO/CMAP transcriptional signatures was performed again using the GRS analysis.

### Datasets used in the analyses

The description, location and processing of the ChIP-seq and gene expression datasets are provided in supplemental methods (Text S1).

### Computational methods

All computational methods are implemented in the R package *treg* which can be downloaded from our web site (http://GenomicsPortals.org). The package also contains processed ChIP-seq data for ERα [10], E2f1 and 15 other transcription factors [32], as well as TREG signatures for ERα and E2f1, and transcriptional signatures derived from GEO GDS datasets and CMAP drug signatures. We have previously described derivation of CMAP signatures [31]. All functional enrichment analyses were performed using the LRpath methodology [58] as implemented in the R package CLEAN [59].

## Supporting Information

Figure S1 **Relative statistical significance of the association between ChIP-seq and differential gene expression data for different window sizes and for different summaries of peak intensities.** The ratio of −log_10_(p-value of enrichment) of differentially expressed genes (FDR<0.1) among genes with high simple scores (MPI, UWS, LWS), and −log_10_(p-value of enrichment) of differentially expressed genes among genes with high TREG binding scores. Red dots correspond to MPI scores, blue dots to UWS scores, and the horizontal blue line corresponds to significance attained by the LWS score. A) The ratios related to E2f1 ChIP-seq data and E2 differential gene expression profile. B) The ratios related to ERα ChIP-seq data and E2+CHX differential gene expression profile. Ratios smaller than 1 indicate higher significance of enrichment when using TREG scores.
(TIF)

Figure S2 **Empirical distribution functions of p-values for four GRS concordance analysis between differential gene expression profiles (E2+CHX and E2) and all 494 ENCODE TREG binding profiles.** For each case, 1000 GRS analyses were performed by first randomly permuting gene labels in one of the profiles. All Empirical Cumulative Distribution Functions (ECDF) of resulting p-values lie at or below the 45 degree line p-values<0.5, indicating strict control of Type I error rates. For 11 ENCODE profiles the GRS was especially conservative (blue lines). The examination of these 11 TREG profiles indicated unusually small number of peaks indicating that in such situations GRS is particularly conservative. A) Empirical distribution functions of p-values for four GRS analyses described in this Table 2. B) E2+CHX differential gene expression profiles vs ENCODE TREG binding profiles. C) E2 differential gene expression profiles vs ENCODE TREG binding profiles.
(TIF)

Figure S3 **GRS vs simple thresholding to assess correlation between TREG binding scores and differential gene expression profiles.** To compare the ability of GRS and the simple thresholding to detect concordance between TREG binding signatures and differential gene expression signatures, we systematically removed genes with strongest TREG binding scores from the E2f1 binding profile and gene expression profiles, and calculated p-values of the GRS and the thresholding analysis in such reduced datasets. The x axes represents the number of remaining genes in the “regulated” group. Red dots represent statistical significance of GRS analysis and blue dots statistical significance of the “thresholding” analysis. These results indicate that the GRS analysis will likely have higher sensitivity when the “concordance signal” between binding and expression data is low, that is when few genes (<1,000) have the TREG binding probability >0.95, while enrichment analysis of “regulated” genes will provide higher statistical significance when the signal is strong (>1,000 genes with TREG probability>0.95) such as it was the case with E2f1. This indicate that it is rational to use GRS as the default method since when the signal is strong, the outcome will not change depending on which method is used, and when the signal is weak, GRS has a higher chance of detecting it.
(TIF)

Figure S4 **Proportion of ENCODE TREG profiles enriched for genes associated with the Cell cycle GO term at a specific statistical significance cut-off (x-axis).** For TREG profiles (TREG) the analysis was performed using logistic regression modeling of the probability of membership in the Cell Cycle gene list based on the TREG scores as implemented in LRpath methodology. For the binding peaks data (Peak), we first established the list of genes with a significant peak within (−10 kb,+10 kb) window around the gene's TSS. Then use Fisher's exact test to calculate statistical significance of the overlap with the Cell cycle gene list. While this approach seems to be somewhat inefficient, it still recapitulates conclusions of TREG analysis that a large proportion of ENCODE binding profiles are enriched for Cell cycle genes.
(TIF)

Figure S5 **Number of peaks in ENCODE profiles for profiles with unusually conservative GRS analysis (blue lines in Fig. S1).**
(TIF)

Table S1 **ERα and E2F1TREG binding scores, gene specific concordance statistic, and TREG concordance scores for all genes.**
(XLSX)

Table S2 **Results of the LRpath enrichment analysis of ERα and E2F1 TREG signatures for all GO terms.**
(XLSX)

Table S3 **Results of the concordance analysis between E2+CHX and E2 differential gene expression profiles and all ENCODE TREG binding profiles.**
(XLSX)

Table S4 **Results of the concordance analysis between TREG ERα up-regulation signature and disease-associated differential gene expression profiles.**
(XLSX)

Table S5 **The genes with gene-specific concordance in the TREG ERα up-regulation signature (p-value(e_g_) <0.001), used for plotting heatmaps in** **Fig. 8** **.**
(XLSX)

Text S1 **Supplemental results, discussion and methods.** Results provide additional results, discussion and methods including the statistical properties of GRS methodology and detailed discussion of the EM algorithm used to estimate parameters of the mixture models.
(DOCX)
