# Supplementary figures and images for: Genome-Wide Signatures of Transcription Factor Activity: Connecting Transcription Factors, Disease, and Small Molecules
Source: PLoS Comput Biol. 2013 Sep 5;9(9):e1003198. doi: 10.1371/journal.pcbi.1003198 (PMC3764016; doi:10.1371/journal.pcbi.1003198)

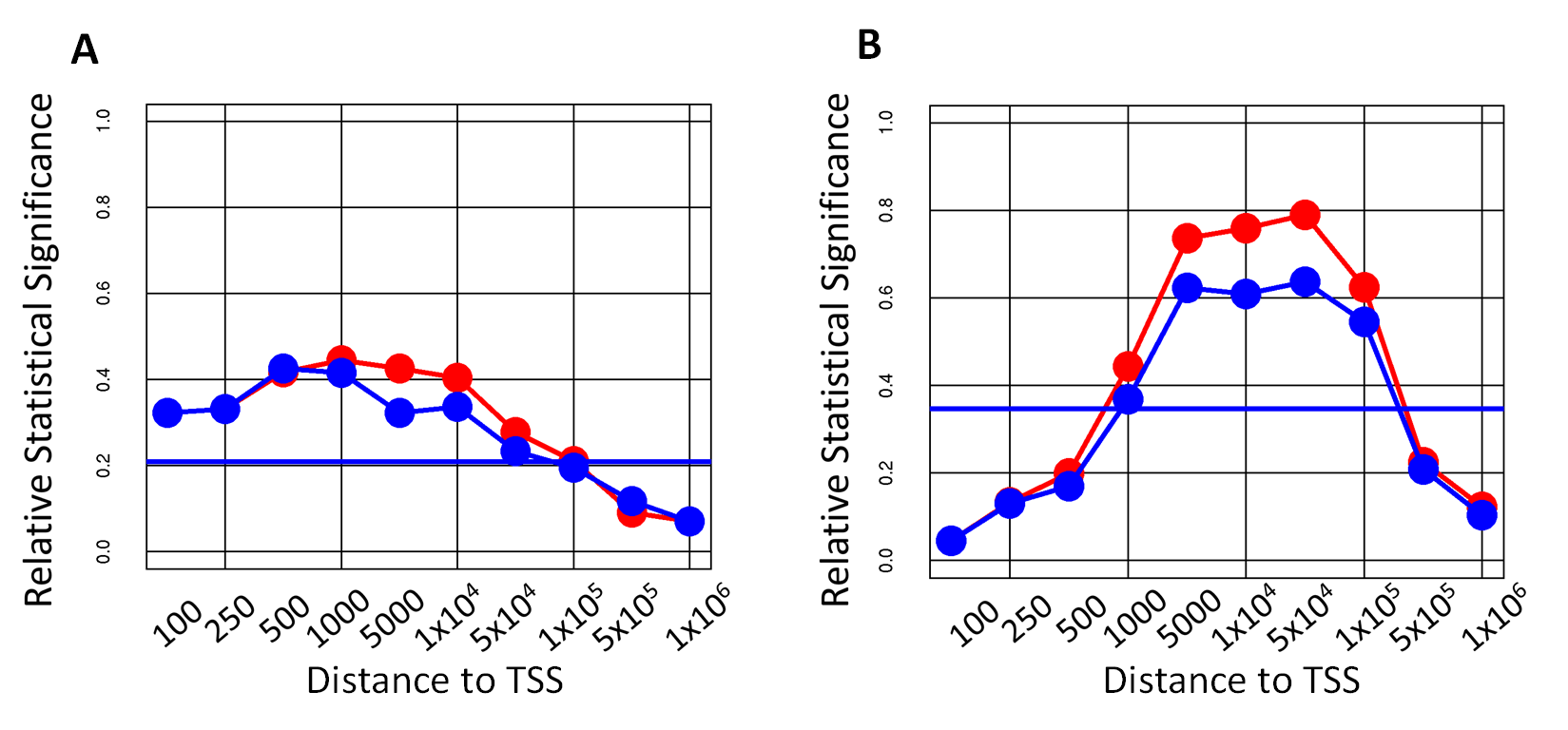

Supplement: Figure S1 — Relative statistical significance of the association between ChIP-seq and differential gene expression data for different window sizes and for different summaries of peak intensities. The ratio of −log10(p-value of enrichment) of differentially expressed genes (FDR<0.1) among genes with high simple scores (MPI, UWS, LWS), and −log10(p-value of enrichment) of differentially expressed genes among genes with high TREG binding scores. Red dots correspond to MPI scores, blue dots to UWS scores, and the horizontal blue line corresponds to significance attained by the LWS score. A) The ratios related to E2f1 ChIP-seq data and E2 differential gene expression profile. B) The ratios related to ERα ChIP-seq data and E2+CHX differential gene expression profile. Ratios smaller than 1 indicate higher significance of enrichment when using TREG scores. (TIF) [file pcbi.1003198.s001.tif]

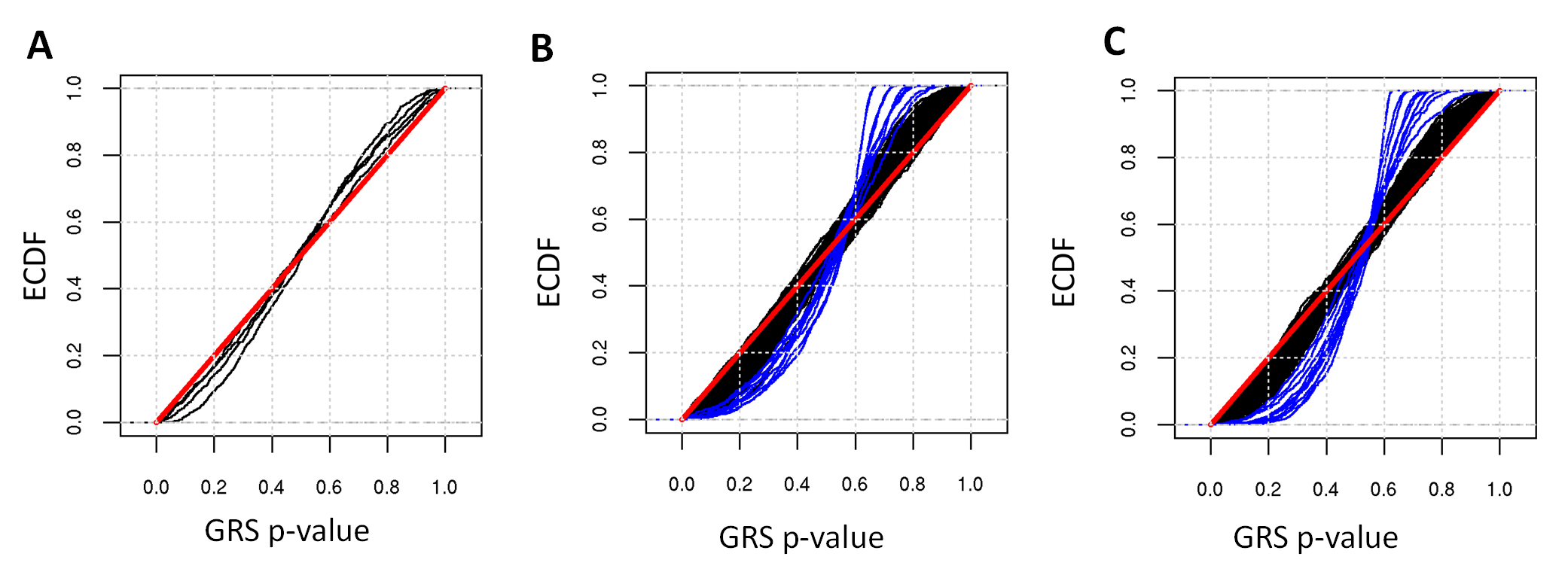

Supplement: Figure S2 — Empirical distribution functions of p-values for four GRS concordance analysis between differential gene expression profiles (E2+CHX and E2) and all 494 ENCODE TREG binding profiles. For each case, 1000 GRS analyses were performed by first randomly permuting gene labels in one of the profiles. All Empirical Cumulative Distribution Functions (ECDF) of resulting p-values lie at or below the 45 degree line p-values<0.5, indicating strict control of Type I error rates. For 11 ENCODE profiles the GRS was especially conservative (blue lines). The examination of these 11 TREG profiles indicated unusually small number of peaks indicating that in such situations GRS is particularly conservative. A) Empirical distribution functions of p-values for four GRS analyses described in this Table 2. B) E2+CHX differential gene expression profiles vs ENCODE TREG binding profiles. C) E2 differential gene expression profiles vs ENCODE TREG binding profiles. (TIF) [file pcbi.1003198.s002.tif]

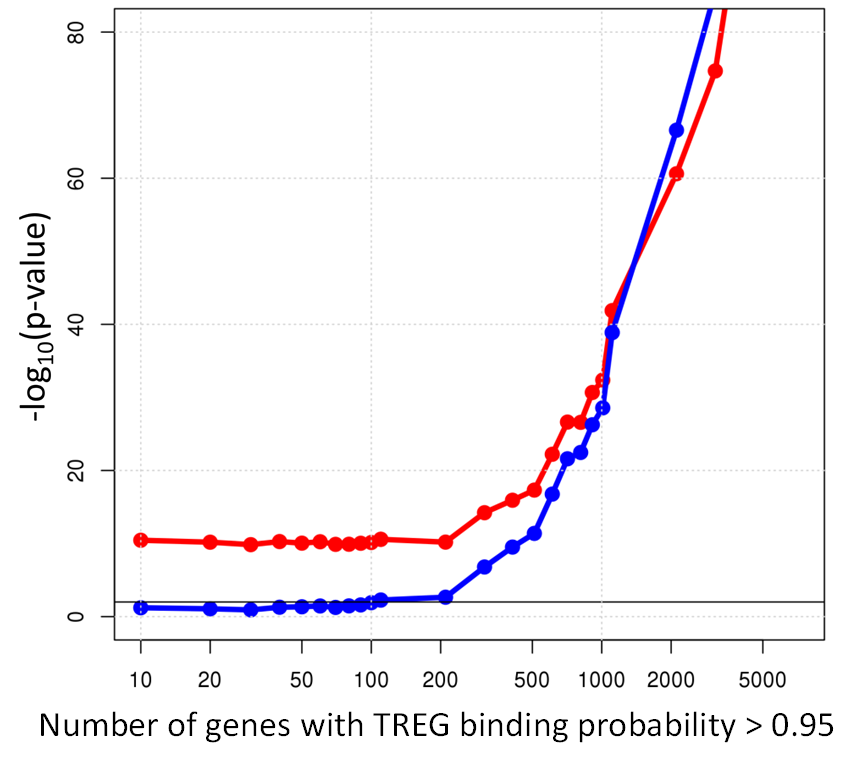

Supplement: Figure S3 — GRS vs simple thresholding to assess correlation between TREG binding scores and differential gene expression profiles. To compare the ability of GRS and the simple thresholding to detect concordance between TREG binding signatures and differential gene expression signatures, we systematically removed genes with strongest TREG binding scores from the E2f1 binding profile and gene expression profiles, and calculated p-values of the GRS and the thresholding analysis in such reduced datasets. The x axes represents the number of remaining genes in the “regulated” group. Red dots represent statistical significance of GRS analysis and blue dots statistical significance of the “thresholding” analysis. These results indicate that the GRS analysis will likely have higher sensitivity when the “concordance signal” between binding and expression data is low, that is when few genes (<1,000) have the TREG binding probability >0.95, while enrichment analysis of “regulated” genes will provide higher statistical significance when the signal is strong (>1,000 genes with TREG probability>0.95) such as it was the case with E2f1. This indicate that it is rational to use GRS as the default method since when the signal is strong, the outcome will not change depending on which method is used, and when the signal is weak, GRS has a higher chance of detecting it. (TIF) [file pcbi.1003198.s003.tif]

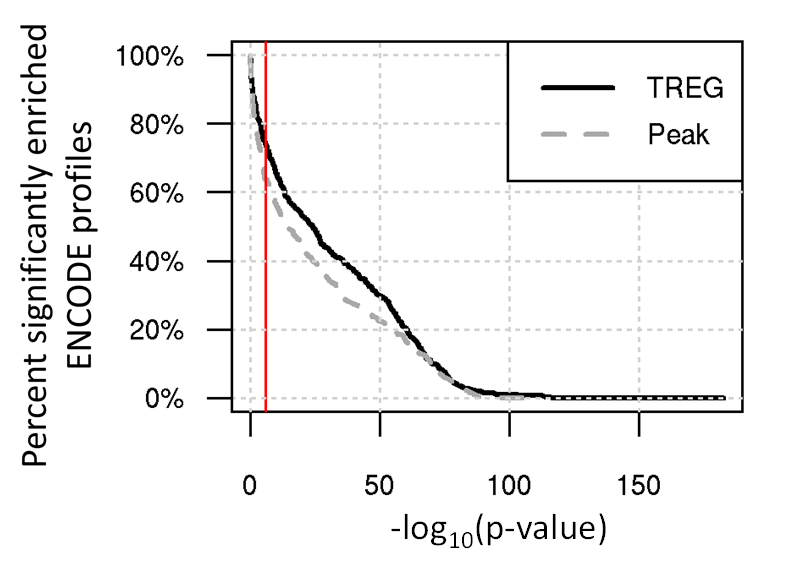

Supplement: Figure S4 — Proportion of ENCODE TREG profiles enriched for genes associated with the Cell cycle GO term at a specific statistical significance cut-off (x-axis). For TREG profiles (TREG) the analysis was performed using logistic regression modeling of the probability of membership in the Cell Cycle gene list based on the TREG scores as implemented in LRpath methodology. For the binding peaks data (Peak), we first established the list of genes with a significant peak within (−10 kb,+10 kb) window around the gene's TSS. Then use Fisher's exact test to calculate statistical significance of the overlap with the Cell cycle gene list. While this approach seems to be somewhat inefficient, it still recapitulates conclusions of TREG analysis that a large proportion of ENCODE binding profiles are enriched for Cell cycle genes. (TIF) [file pcbi.1003198.s004.tif]

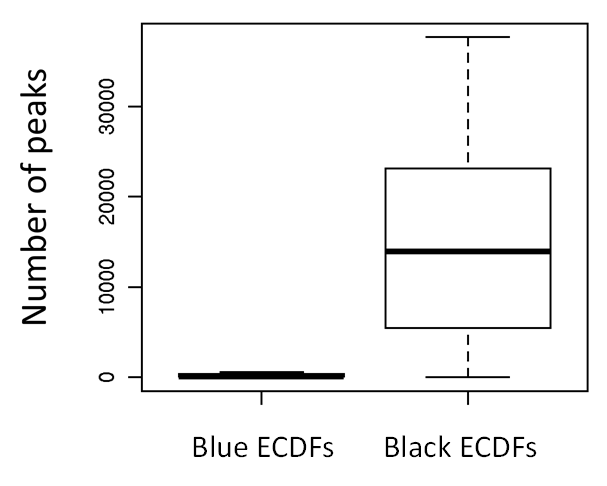

Supplement: Figure S5 — Number of peaks in ENCODE profiles for profiles with unusually conservative GRS analysis (blue lines in Fig. S1). (TIF) [file pcbi.1003198.s005.tif]
