# Supplementary material for: Genome-Wide Signatures of Transcription Factor Activity: Connecting Transcription Factors, Disease, and Small Molecules
Source: PLoS Comput Biol. 2013 Sep 5;9(9):e1003198. doi: 10.1371/journal.pcbi.1003198 (PMC3764016; doi:10.1371/journal.pcbi.1003198)
Supplement: Text S1 — Supplemental results, discussion and methods. Results provide additional results, discussion and methods including the statistical properties of GRS methodology and detailed discussion of the EM algorithm used to estimate parameters of the mixture models. (DOCX) [file pcbi.1003198.s011.docx]

**Supplemental Results, Discussion and Methods for manuscript:**

**Genome-wide signatures of transcription factor activity: connecting transcription factors, disease, and small molecules**

Jing Chen, Zhen Hu, Mukta Phatak, John Reichard, Johannes M Freudenberg, Siva Sivaganesan and Mario Medvedovic

**Supplemental Results**

**Comparison of TREG binding scores to simple summaries (Fig S1):**  We assessed the effectiveness of the TREG binding score by comparison to three simple scoring methods, the maximum peak intensity (MPI) (results presented in the main text) within a window of specific size around TSS, the unweighted sum of intensities (UWS) within a window of specific size around TSS, and the linearly weighted sum (LWS) within the window [-50kb, 50kb] around TSS where weights were a linear function of the distance starting with 1 for zero distance and being 0 for 50kb distance. The three scores were evaluated by comparing the enrichment of genes with high evidence of TF binding among genes differentially expressed in appropriately matched experiments (same as in main text). Fig S1 shows the relative levels of enrichment for the three scores (MPI, UWS, LWS) in comparison to the TREG binding score. MPI and UWS were compared across a range of window sizes around TSSs. Simple scores (MPI and UWS) never attain the level of statistical significance of enrichment attained by TREG binding scores and exhibit the same TF-specificity as discussed in main text. LWS scores perform worse than the MPI and UWS for optimal window sizes, and it performs significantly worse than TREG binding scores. This confirms conclusions from the main text that TREG binding scores provide the best correlation with expression changes for simple unweighted and weighted scores that can be derived from ChIP-seq data.

**GRS comparisons strictly controls Type I error rates:** To demonstrate that GRS is producing expected distribution of p-values under the null hypothesis we examined empirical cumulative distribution functions (ECDFs) of p-values after randomly permuting gene labels in TREG binding profile before GRS analysis (Fig S2). ECDFs for all four GRS analyses in Table 2 (Fig S2A) lie below the 45 degree line for p-values<0.5 indicating strict control of Type I error rates. The same conclusion was reached for GRS analysis with ENCODE TF binding (Fig S2B and S2C).

**GRS vs simple thresholding to assess concordance between TREG binding scores and differential gene expression profiles:** In the context of gene expression data analysis, the GRS approach showed an increase in statistical power over methods requiring significance thresholds and it was shown that it is impossible to establish universally “good” thresholds for performing such analysis [1]. To test these results in the context of concordance analysis between differential gene expression profiles and TREG binding profiles, we repeated the analysis by first assigning genes into “regulated” and “non-regulated” groups based on the TREG probability. A gene was placed in the “regulated” group if the corresponding TREG probability (p_g_) was greater than 0.95. Then we performed a simple enrichment analysis of differentially expressed genes in the “regulated” group using LRpath [2]. Results of these analyses (Table below) were similar to results using GRS (maint text Table 2) indicating that thresholding on TREG probability may be equally appropriate analytical strategy in this setting.

| **Transcription Factor** | **E2** | **E2 + CHX** |
| --- | --- | --- |
| **E2f1** | 1.4x10^-192^ | 3.6x10^-13^ |
| **ERα** | 1.6x10^-25^ | 4.3x10^-90^ |

However, based on our experience with the expression data, we expected that the advantages of GRS in this context would transpire when the “concordance signal” in the ChIP-seq and expression data is relatively weak [1]. To test this conjecture, we systematically removed genes with highest TREG binding scores from the E2f1 binding and gene expression profiles and compared p-values of the GRS and the thresholding analysis in such reduced datasets (Fig S3). These results indicate that the GRS analysis will likely have higher sensitivity when the “concordance signal” between binding and expression data is low (<1,000 genes with TREG binding probability > 0.95), while simple thresholding would result in higher statistical significance when the signal is strong (>1,000 genes with TREG binding probability > 0.95), such as it was the case with E2f1 data (7,907 genes with TREG binding probability of >0.95). These results are consistent with our ERα results where there were 973 genes with TREG binding probability > 0.95 and GRS produced higher statistical significance than the thresholding approach. Our results also indicate that for even weaker signals (<200 genes with TREG binding probability > 0.95) GRS would be able to detect the concordance while the thresholding approach would not. This indicates that GRS is the rational choice to be used in such an analysis as the default method because when the signal is strong, the outcome will not change depending on which method is used, and when the signal is weak, GRS has a higher chance of detecting it.

**Finding evidence of ERα activity in complex transcriptional profiles:** In the main text we show a dramatic improvement in sensitivity to detect ERα activity in complex transcriptional signatures. It is important to point out that E2+CHX differential gene expression signature exhibits even higher statistical significance of concordance with both DRGEPs than the ERα TREG signature (-log10(p-value) of >200 and 34 for E2 and ER-/+ respectively). This is most likely due to incomplete suppression of the proliferation-related secondary ERα targets in the E2+CHX experiment. For example, it has been previously shown in this same system that up-regulation of the PCNA gene is not a consequence of direct binding ERα and it depends on de novo protein synthesis [3]. Yet PCNA expression is elevated in E2+CHX profile (p-value=2x10^-4^). PCNA gene and protein are commonly used proliferation markers. Consequently, the concordance between transcriptional signatures is driven to some extend by indirect ERα targets. Because the objective of the analysis is to establish regulatory activity of the ERα concordance with E2+CHX transcriptional signature is not an appropriate method to do so.

**Mining for ERα activity in perturbation signatures and disease-related gene expression profiles:** We searched for disease-associated GEO profiles showing evidence of ERα regulatory activity. Top 10 identified transcriptional profiles (Table below) list diseases for which there has been clear evidence of ERα regulatory activity such as breast cancer subtypes (GDS2250 [4], GDS1329 [5]) ovarian endometriosis (GDS2835 [6]), and cervical cancer (GDS3233 [7]). For these diseases, the ERα activity revealed by TREG analysis is concordant with the differential expression of the gene encoding ERα (Table 4). In other implicated disease-associated transcriptional profiles, there was no evidence of differential expression of ERα. Progression of Barrett's metaplasia to adenocarcinoma is associated with the suppression of the transcriptional programs of epidermal differentiation (GDS1231 [8]) which is concordant with our functional analysis of ERα signatures. Differences in myelodysplastic syndromes (GDS2118 [9] have not been previously associated with differences in ERα regulatory activity. However, the incidence of different lymphoma subtypes has been previously associated with differences in reproductive hormone exposure [10]. On the other hand, epigenetic modulation of ERα activity has been implicated in the pathophysiology of myelodysplastic syndromes [11]. The additional evidence provided by our analysis may be important in studies of the role of ERα in these diseases. There was no obvious connection between ERα regulation and differences between lung adenocarcinomas and squamous cell carcinomas (GDS3627 [12]). Results for all disease-associated GEO profiles are given in the Table S4.

**Table:** Top 10 disease-related differential gene expression profiles based on the concordance with the TREG signatures of ERα regulatory activity. An upward arrow (**↑**) next to the p-value in the **ERα signature** column indicates statistically significantly increased expression of ERα regulated genes in the first vs second sample type (eg in basal-like cancer **vs**. non-basal-like cancer comparisons, this would indicate increased expression in basal-like cancer samples), and a downward arrow (**↓**) indicates statistically significantly decreased expression of ERα regulated genes. P-values and arrows in the **ERα expression** columns indicated the statistical significance of the difference in expression of the ERα gene (ESR1). The absence of an arrow indicates no statistical significance of differences in expression levels.

| **Dataset** | **Comparison** | **ERα signature** | **ERα expression** |
| --- | --- | --- | --- |
| GDS2250 | basal-like cancer **vs.** non-basal-like cancer | **↓**8.77*10^-21^ | **↓**1.71*10^-03^ |
| GDS1780 | lymph node metastasis **vs.** primary tumor | **↓**1.26*10^-18^ | 3.21*10^-01^ |
| GDS2835 | normal **vs**. ovarian endometriosis | **↑**1.52*10^-17^ | **↑**6.41*10^-09^ |
| GDS1321 | adenocarcinoma **vs**. Barrett's esophagus | **↓**1.07*10^-13^ | 5.75*10^-01^ |
| GDS3627 | adenocarcinoma **vs**. squamous cell carcinoma | **↓**9.16*10^-12^ | 1.10*10^-01^ |
| GDS1329 | basal tumor **vs**. luminal tumor | **↓**1.03*10^-11^ | **↓**8.39*10^-05^ |
| GDS2118 | Ref anemia excess blasts **vs**. ref anemia ringed sideroblasts | **↓**1.48*10^-11^ | 3.33*10^-01^ |
| GDS1329 | apocrine tumor **vs**. luminal tumor | **↓**2.17*10^-09^ | **↓**2.81*10^-03^ |
| GDS756 | metastasic tumor **vs**. primary tumor | **↓**3.04*10^-09^ | 2.33*10^-01^ |
| GDS3233 | cervical cancer primary tumor **vs**. normal | **↓**1.44*10^-08^ | **↓**1.13*10^-06^ |

**Analysis of ENCODE TF binding profiles:** ENCODE ChIP-seq data was downloaded directly from the Genome Browser MySQL database by selecting TF-related “Peaks” tables. When experimental replicates were available, TREG analysis was performed for each replicate separately and final TREG binding scores and probabilities were constructed by averaging scores and probabilities for experimental replicates. This analysis has ultimately yielded 494 unique TREG profiles across all TF by cell line by treatment combinations. The GRS analysis between our two transcriptional signatures (E2+CHX and E2) and ENCODE signatures yielded many statistically significant concordances (especially E2 signatures). We first confirmed that GRS indeed correctly controls Type I error rates in these comparisons (Fig S2). A closer inspection of ENCODE signatures showed that most of them are indeed enriched by proliferation-related genes which explains preponderance of statistically significant concordances with E2 expression signature (Fig S4). ENCODE profiles with particularly conservative ECDFs (blue lines in Fig S2) turned out to have relatively few peaks in comparisons to other ENCODE profiles (Fig S5).

**Supplemental Discussion**

**Expending TREG methodology to incorporate epigenetic data:** Our methods are complementary to methods used to analyze the recently released ENCODE project data [13,14]. For some experimental conditions, the ENCODE project provides additional data types that can be used in assessing the functionality of TF binding peaks, such as distribution of specific epigenetic histone modifications. Methodologically, statistical models underpinning TREG framework can be extended to incorporate epigenetic ChIP-seq data when available, possibly leading to more precise signatures of transcriptional regulation. For example, sophisticated statistical models have been developed to predict gene expression levels within a single well-defined biological system (developing drosophila embryo) by integrating data on multiple TFs and epigenetic modifications [15]. While generalizability of the derived model to different systems, (eg human disease gene expression data) is not clear and has not been tested, the Bayesian network-based model used by Wilczynski et al [15] for assessing the activity of enhancers is appealing and may be a natural way to integrate multiple data types within the TREG framework. The major objective of the TREG framework is to derive signatures that are generalizable beyond the biological system that was used to generate data*.* It is likely that signatures derived using such integrative models would provide better description of regulatory mechanisms in the biological systems from which data is generated. However such more precise models could end up modeling too closely the idiosyncrasies of the model system in which data is generated which could adversely affect generalizability of derived signatures. Therefore, the scope of such integrative strategies in this context still needs to be assessed.

**Supplemental Methods**

**Datasets used in the analyses:** Processed ERα [16] and E2f1 [17] ChIP-seq data in the form of peak locations and peak intensities was downloaded from GEO (GSE14664 and GSE11431). For the mouse E2f1 data, TREG binding scores were mapped to appropriate human homologs using the Homologene mappings. Raw gene expression data for the estradiol-treated MCF-7 cell line experiment [18] and breast cancer contrasting ER+ and ER- tumors [19] was downloaded from GEO (GSE8597 and GSE3494). Data was processed by performing background correction, quantile normalization, and calculation of expression set summaries using the Robust Multichip Average (RMA) protocol [20] as implemented in the *affy* Bioconductor [21] package. Gene-specific probesets were constructed using the Entrez Gene – centric definitions [22]. Differential gene expression profiles were established using Empirical Bayes linear models as implemented in *limma* Bioconductor package [23]. For the creation of ER+ and ER- breast cancer differential gene expression profiles, the normalized data was downloaded from GEO (GSE2740 [24]) and differential gene expression analysis was again performed using Empirical Bayes linear models. The construction of the GEO and Connectivity Map [25] transcriptional signatures was previously described in [1].

**The choice of probability distributions in the normal-exponential mixture model:** When postulating this mixture model, our goal was to use the simplest possible model capable of distinguishing between two different populations of scores. The distributions used (normal and exponential) are reasonable approximations for the two types of scores “functional” and” non-functional”, based on their commonly accepted characteristics and are useful in separating these two distributions of scores. Theoretically, the “functional” scores will be a weighted sum of multiple peaks sufficiently close to the TSS to have non-zero weights, which is expected to be bell-shaped. On the other, hand non-functional peaks will be based on sporadic occurrence of peaks anywhere within the large 2MB windows. Such sporadic peaks are likely to be of low intensity and they are likely to occur at distances that are heavily down-weighted in our first-stage model. Exponential distribution is the simplest model that captures the behavior of such non-functional scores that are peaked around zero and have diminishing probability of being greater than zero. Our empirical examinations of the score distributions supported these assumptions

**Convergence of the EM algorithm:**  The EM algorithm used to estimate parameters of the mixture models is a “hill-climbing” algorithm whose convergence may depend on the initial values of the parameters. Due to the complex likelihood function, the convergence issues can be particularly severe in the case of mixture models [26]. To side-step potential problems with algorithm converging to different solutions over multiple starts we initialize model parameters with pre-defined values which are either chosen to represent reasonable levels in general (exponential-uniform mixture) or are estimated from the data (exponential-normal mixture). The objective of our algorithm is to find the local maxima of the likelihood function in the neighborhood of such reasonable starting points. This general strategy has been used in other applications of the EM algorithm in the context of mixture modeling. For example, the gold-standard MCLUST methodology for fitting finite mixture models [27,28] uses hierarchical clustering to come-up with a “good” starting point for the EM algorithm and then EM algorithm is used to improve upon the such a reasonable starting point. In our tests, deviations within reasonable range around these values (doubling and halving of parameters) did not affect the parameter estimates to which algorithm converged. This indicates that the selected initial values are not near local minima for the likelihood function and parameters estimates are reasonably robust with respect to varying the starting points. Following are specific initialization values for mixture parameters and test levels for which EM converged to the same estimates. Tests were performed for the ERα ChIP-seq data. The first row in each table corresponds to initial parameters used (red) and each perturbed parameter in subsequent rows is blue:

**Exponential-uniform mixture model (main text Fig 1B)**

| **Initial values (π_0_, λ_0_)** | **Estimated values (π, λ)** |
| --- | --- |
| **(0.1, 0.1)** | **(0.002818247, 0.001530033)** |
| (0.05, 0.1) | (0.002815981, 0.001532684) |
| (0.2, 0.1) | (0.002817703, 0.001530669) |
| (0.1, 0.2) | (0.002816453, 0.001532132) |
| (0.1,0.05) | (0.00281663, 0.001531924) |

**Exponential-normal mixture model (main text Fig 1C)**

In this model, we use a combination of heuristic estimation of the parameter for the exponential component and the EM algorithm for the estimation of other parameters in the mixture model. The exponential component parameter *ψ* is estimated by calculating the maximum score supported by data points, *τ*, under the assumption of uniform distribution and then setting *ψ* so that *τ* is the 0.999^th^ quantile of the corresponding exponential distribution (ie $\int_{\tau}^{\infty} f_{E}\left( x,\psi\right)dx=0.001$). The score *τ* is calculated by first establishing the maximum number of peaks for a gene (*m*). The [0, 10^6^] interval is then split into m equal-sized subintervals *w_k_=[(k-1)*10^6^/m,k*10^6^/m], k=1,…,m.* The new set of peaks is created by calculating peak intensities as averages of all peaks within the distance *w_k_* to any gene for each interval *w_k_* and assigning the distance to TSS as the distance from the center of intervals *w_k_*. *τ* is then calculated as the weighted average of such peaks using the exponential-uniform mixture model.

Given *ψ*, the mean of the normal distribution is initialized as *μ_0_=τ*+1. That is, the initial mean for the normal component was shifted to the right by 1 from the maximum binding score expected under uniform distribution of peaks in the current dataset. For the ERα ChIP-seq data *τ=0.32 and*  *ψ=* 21.78. The robustness with respect to the initial values for the mean of the normal component (*μ_0_*) was assessed by changing the shift from *τ* to 0.5 or 2.

| **Initial values (1-η_0_, ψ_0_, μ_0_, σ_0_)** | **Estimated values (1-η, ψ, μ, σ)** |
| --- | --- |
| (0.5, 21.78, 1.32, 1) | (0.9432077, 21.78556, 2.426446, 1.269299) |
| (0.25, 21.78, 1.32, 1) | (0.9432091, 21.78556, 2.426499, 1.26922) |
| (0.9, 21.78, 1.32, 1) | (0.943209, 21.78556, 2.426493, 1.269228) |
| (0.5, 21.78, 2.32, 1) | (0.9432088, 21.78556, 2.426486, 1.269238) |
| (0.5, 21.78,0.82, 1) | (0.9432072, 21.78556, 2.426426, 1.269329) |
| (0.5, 21.78, 1.32, 2) | (0.9432078, 21.78556, 2.426449, 1.269295) |
| (0.5, 21.78, 1.32, 0.5) | (0.9432087, 21.78556, 2.426484, 1.269242) |

**Integrating TREG binding profile and differential gene expression to identify truly regulated genes:** Identifying genes that both have high probability of “functional” TF binding and are differentially expressed is complicated by the need to set arbitrary thresholds for statistical significance. We apply the GRS framework to assess the concordance between the TREG binding profile and the differential gene expression profile, and to identify genes with statistically significant concordance. Suppose that for each gene *g*=1,…,*G* we are given the TREG binding score $S_{g}^{T}$, the associated probability of functional binding $p_{g}^{T}$, -log of differential gene expression p-value $S_{g}^{D}$and the associated posterior probability of differential expression$p_{g}^{D}$. We define the global TREG concordance statistics *E* (Fig 1 **Eq 6**), and use the asymptotic null distribution derived under the assumption of no concordance [1]. The gene’s TREG concordance statistics *e_g_* measures the contribution of an individual gene to the overall concordance (Fig 1 **Eq7**). The statistical significance of *e_g_*’s is assessed by comparing it to the null distribution of such scores obtained by randomly permuting gene labels of either TREG binding scores of differential gene expression data.

References

Reference List

1. Freudenberg JM, Sivaganesan S, Phatak M, Shinde K, Medvedovic M (2011) Generalized random set framework for functional enrichment analysis using primary genomics datasets. Bioinformatics 27: 70-77.

2. Sartor MA, Leikauf GD, Medvedovic M (2009) LRpath: a logistic regression approach for identifying enriched biological groups in gene expression data. Bioinformatics 25: 211-217. btn592 [pii];10.1093/bioinformatics/btn592 [doi].

3. Wang C, Yu J, Kallen CB (2008) Two Estrogen Response Element Sequences Near the <italic>PCNA</italic> Gene Are Not Responsible for Its Estrogen-Enhanced Expression in MCF7 Cells. PLoS ONE 3: e3523.

4. Richardson AL, Wang ZC, De Nicolo A, Lu X, Brown M et al. (2006) X chromosomal abnormalities in basal-like human breast cancer. Cancer Cell 9: 121-132.

5. Farmer P, Bonnefoi H, Becette V, Tubiana-Hulin M, Fumoleau P et al. (2005) Identification of molecular apocrine breast tumours by microarray analysis. 24: 4660-4671.

6. Hever A, Roth RB, Hevezi P, Marin ME, Acosta JA et al. (2007) Human endometriosis is associated with plasma cells and overexpression of B lymphocyte stimulator. PNAS 104: 12451-12456.

7. Kwasniewska A, Postawski K, Gozdzicka-Jozefiak A, Kwasniewski W, Grywalska E et al. (2011) Estrogen and progesterone receptor expression in HPV-positive and HPV-negative cervical carcinomas. Oncol Rep 26: 153-160. 10.3892/or.2011.1256 [doi].

8. Kimchi ET, Posner MC, Park JO, Darga TE, Kocherginsky M et al. (2005) Progression of Barrett's Metaplasia to Adenocarcinoma Is Associated with the Suppression of the Transcriptional Programs of Epidermal Differentiation. Cancer Res 65: 3146-3154.

9. Pellagatti A, Cazzola M, Giagounidis AAN, Malcovati L, Porta MGD et al. (2006) Gene expression profiles of CD34+ cells in myelodysplastic syndromes: involvement of interferon-stimulated genes and correlation to FAB subtype and karyotype. Blood 108: 337-345.

10. Lee JS, Bracci PM, Holly EA (2008) Non-Hodgkin Lymphoma in Women: Reproductive Factors and Exogenous Hormone Use. American Journal of Epidemiology 168: 278-288.

11. Warlick ED, Smith BD (2007) Myelodysplastic syndromes: review of pathophysiology and current novel treatment approaches. Curr Cancer Drug Targets 7: 541-558.

12. Kuner R, Muley T, Meister M, Ruschhaupt M, Buness A et al. (2009) Global gene expression analysis reveals specific patterns of cell junctions in non-small cell lung cancer subtypes. Lung Cancer 63: 32-38. S0169-5002(08)00193-1 [pii];10.1016/j.lungcan.2008.03.033 [doi].

13. 2012) An integrated encyclopedia of DNA elements in the human genome. Nature 489: 57-74.

14. Cheng C, Alexander R, Min R, Leng J, Yip KY et al. (2012) Understanding transcriptional regulation by integrative analysis of transcription factor binding data. Genome Res 22: 1658-1667.

15. Wilczynski B, Liu YH, Yeo ZX, Furlong EEM (2012) Predicting Spatial and Temporal Gene Expression Using an Integrative Model of Transcription Factor Occupancy and Chromatin State. PLoS Comput Biol 8: e1002798.

16. Welboren WJ, van Driel MA, Janssen-Megens EM, van Heeringen SJ, Sweep FC et al. (2009) ChIP-Seq of ER[alpha] and RNA polymerase II defines genes differentially responding to ligands. EMBO J advanced online publication.

17. Chen X, Xu H, Yuan P, Fang F, Huss M et al. (2008) Integration of External Signaling Pathways with the Core Transcriptional Network in Embryonic Stem Cells. Cell 133: 1106-1117.

18. Bourdeau V, Deschenes J, Laperriere D, Aid M, White JH et al. (2008) Mechanisms of primary and secondary estrogen target gene regulation in breast cancer cells. Nucl Acids Res 36: 76-93.

19. Miller LD, Smeds J, George J, Vega VB, Vergara L et al. (2005) From The Cover: An expression signature for p53 status in human breast cancer predicts mutation status, transcriptional effects, and patient survival. PNAS 102: 13550-13555.

20. Irizarry RA, Hobbs B, Collin F, Beazer-Barclay YD, Antonellis KJ et al. (2003) Exploration, normalization, and summaries of high density oligonucleotide array probe level data. Biostatistics 4: 249-264.

21. Gentleman RC, Carey VJ, Bates DM, Bolstad B, Dettling M et al. (2004) Bioconductor: open software development for computational biology and bioinformatics. Genome Biol 5: R80.

22. Dai M, Wang P, Boyd AD, Kostov G, Athey B et al. (2005) Evolving gene/transcript definitions significantly alter the interpretation of GeneChip data. Nucl Acids Res 33: e175.

23. Smyth GK (2005) Limma: linear models for microarray data. In: Gentleman R, Carey V, Dudoit S, Irizarry R, Huber W, editors. Bioinformatics and Computational Biology Solutions using R and Bioconductor. New York: Springer. pp. 397-420.

24. Oh DS, Troester MA, Usary J, Hu Z, He X et al. (2006) Estrogen-Regulated Genes Predict Survival in Hormone ReceptorΓÇôPositive Breast Cancers. Journal of Clinical Oncology 24: 1656-1664.

25. Lamb J, Crawford ED, Peck D, Modell JW, Blat IC et al. (2006) The Connectivity Map: using gene-expression signatures to connect small molecules, genes, and disease. Science 313: 1929-1935.

26. Medvedovic M, Succop P, Shukla R, Dixon K (2001) Clustering mutational spectra via classification likelihood and markov chain monte carlo algorithms. Journal of Agricultural, Biological, and Environmental Statistics 6: 19-37.

27. Fraley C, Raftery AE (1999) Mclust: Software for modelbased cluster analysis. Journal of Classification. Journal of Classification 16: 297-306.

28. Fraley C, Raftery AE (2002) Model-Based Clustering, Discriminant Analysis, and Density Estimation. JASA 97: 611-631.
